# Altered Mental Status: Current Evidence-based Recommendations for Prehospital Care

**DOI:** 10.5811/westjem.2018.1.36559

**Published:** 2018-03-08

**Authors:** Ashley Sanello, Marianne Gausche-Hill, William Mulkerin, Karl A. Sporer, John F. Brown, Kristi L. Koenig, Eric M. Rudnick, Angelo A. Salvucci, Gregory H. Gilbert

**Affiliations:** * Los Angeles County Emergency Medical Services (EMS) Agency, Santa Fe Springs, California; † Harbor UCLA, Department of Emergency Medicine, Torrance, California; ‡ David Geffen School of Medicine, Department of Emergency Medicine, Los Angeles, California; § Stanford University, Department of Emergency Medicine, Stanford, California; ¶ University of California, San Francisco, Department of Emergency Medicine, San Francisco, California; || EMS Medical Directors Association of California; # County of San Diego, Health & Human Services Agency, Emergency Medical Services, San Diego, California; ** University of California, Irvine, Department of Emergency Medicine, Orange, California; †† NorCal EMS Agency, Redding, California; ‡‡ Ventura County EMS Agency, Oxnard, California

## Abstract

**Introduction:**

In the United States emergency medical services (EMS) protocols vary widely across jurisdictions. We sought to develop evidence-based recommendations for the prehospital evaluation and treatment of a patient with an acute change in mental status and to compare these recommendations against the current protocols used by the 33 EMS agencies in the State of California.

**Methods:**

We performed a literature review of the current evidence in the prehospital treatment of a patient with altered mental status (AMS) and augmented this review with guidelines from various national and international societies to create our evidence-based recommendations. We then compared the AMS protocols of each of the 33 EMS agencies for consistency with these recommendations. The specific protocol components that we analyzed were patient assessment, point-of-care tests, supplemental oxygen, use of standardized scoring, evaluating for causes of AMS, blood glucose evaluation, toxicological treatment, and pediatric evaluation and management.

**Results:**

Protocols across 33 EMS agencies in California varied widely. All protocols call for a blood glucose check, 21 (64%) suggest treating adults at <60mg/dL, and half allow for the use of dextrose 10%. All the protocols recommend naloxone for signs of opioid overdose, but only 13 (39%) give specific parameters. Half the agencies (52%) recommend considering other toxicological causes of AMS, often by using the mnemonic AEIOU TIPS. Eight (24%) recommend a 12-lead electrocardiogram; others simply suggest cardiac monitoring. Fourteen (42%) advise supplemental oxygen as needed; only seven (21%) give specific parameters. In terms of considering various etiologies of AMS, 25 (76%) give instructions to consider trauma, 20 (61%) to consider stroke, and 18 (55%) to consider seizure. Twenty-three (70%) of the agencies have separate pediatric AMS protocols; others include pediatric considerations within the adult protocol.

**Conclusion:**

Protocols for patients with AMS vary widely across the State of California. The evidence-based recommendations that we present for the prehospital diagnosis and treatment of this condition may be useful for EMS medical directors tasked with creating and revising these protocols.

## INTRODUCTION

Altered mental status (AMS) represents a broad spectrum of disease processes, making treatment modalities equally broad and varied. If the cause for AMS is found, the prehospital care providers will then transition to that more-specific protocol. However, emergency medical service (EMS) providers have limited time to evaluate these undifferentiated patients. Therefore, guidelines for assessment and initial treatment prior to arriving at an emergency department (ED) are essential. The prevalence of AMS in the prehospital care setting is not well known given the limited research in this area. One California county found 27% of all EMS patients had an abnormal Glasgow Coma Scale (GCS).^1^ ED data report AMS at a prevalence between 1–10% of visits.^2^–^4^ Prehospital protocols and treatment recommendations for AMS vary widely across the U.S.^5^ We provide a summary of available evidence for prehospital assessment and treatment of patients with undifferentiated AMS and additionally evaluate consistency across California protocols.

## METHODS

The State of California divides the EMS system into 33 local EMS agencies (LEMSAs). One set of governmental medical control policies regulates EMS response in each county-wide or region-wide system. Medical directors of those agencies, along with other interested EMS medical directors within the state, make up the EMS Medical Directors Association of California (EMDAC). EMDAC supports and guides the various agencies and makes recommendations to the California EMS Authority about policy, legislation and scope of practice. In an effort to improve the quality of EMS care in our state, EMDAC has endeavored to create evidence-based recommendations for EMS protocols.^2^,^3^ These recommendations are intended to assist medical directors of the LEMSAs to develop high-quality, evidence-based protocols.

A subcommittee of EMDAC developed this manuscript and chose by consensus the elements that should be included in any protocol for a patient found to have AMS by EMS personnel. The subcommittee then created a narrative review of the existing evidence for prehospital treatment of a patient with AMS. Clinical questions regarding those interventions were developed in the PICO (population, intervention, control and outcome) format. In answering these questions, our population consisted of those patients in the prehospital setting with undifferentiated AMS, not those with clear causes for their AMS.

We relied heavily on recommendations made by various organizations that have performed systematic reviews and meta-analyses regarding treatment interventions. We supplemented the recommendations from those organizations with additional literature searches through PubMed from 1966 to 2017 for each question. The initial literature review of PubMed searched for the term “Prehospital and Altered Mental Status.” That yielded 42 articles, only five of which were published in English and pertinent to the topics identified by the EMDAC subcommittee (Figure). This search was supplemented with additional PubMed searches for each clinical question. See Appendix table for additional search terms.

We assigned levels of evidence (LOE) and graded our recommendations based on the American College of Emergency Physicians (ACEP) process of creating clinical policies,^4^ with slight modification, such as the EMDAC committee members performed literature search and assigned classes of evidence to diagnostic, therapeutic and prognostic questions, instead of a professional librarian or methodologist. This committee of EMDAC reviewed studies and assigned LOE based on the study design, including features such as data collection methods, randomization, blinding, outcome measures and generalizability.

LOE I consisted of randomized controlled trials, prospective cohort studies, meta-analysis of randomized trials or prospective studies or clinical guidelines/comprehensive review. LOE II consisted of nonrandomized trials and retrospective studies. LOE III consisted of case series, case reports, and expert consensus. After assigning LOE to the studies, we translated those to clinical grades of recommendations using the following standards:

### Level A Recommendations

- Prehospital recommendations with a strong degree of certainty based on one or more LOE I studies or multiple LOE II studies.

### Level B Recommendations

- Prehospital recommendations with a moderate degree of certainty based on one or more LOE II studies or multiple LOE III studies.

### Level C Recommendations

- Prehospital recommendations based on only poor quality or minimal LOE III studies or based on consensus.

### No Recommendation

- No recommendation was given in those cases where only preliminary data or no published evidence exists and we had no expert consensus.
- We also withheld recommendation when studies, no matter their LOE, showed conflicting data.

After answering the clinical question and providing recommendations for diagnostic and treatment interventions, we reviewed each current AMS protocol from the 33 agencies for consistency with the recommendations. The clinical protocols were reviewed during the months of November 2016 and July 2017.

### Patient Assessment

#### Clinical Question

What patient and scene assessment should be performed by EMS for patients with AMS?

#### Summary of Current Evidence

Patients with an abnormal GCS are more likely to have a history of the condition known to be associated with their confused state, especially alcohol use disorder/hepatic encephalopathy, diabetes, illicit substance use, stroke/transient ischemic attack (TIA) and seizure.^1^ This is particularly true if they have had a history of transient AMS in the past.^5^ Obtaining the patient’s history of present illness and past medical history often leads to identifying the cause of AMS.^6^,^7^

EMS providers have a unique opportunity to obtain pertinent history from family and bystanders who have knowledge of the patient’s underlying medical conditions and access to materials found in the home. Often, if the history does not clarify the cause for AMS, the physical examination and environment will provide the needed clues.^2^,^8^ If evidence as to the etiology of their AMS is found during scene assessment, these findings should be relayed to receiving ED personnel.

Given that neurologic causes (seizures, TIA/stroke), toxicologic causes, hypoglycemia and infection are the most common reasons for AMS, it would be prudent to check for signs of these pathologies. A full examination focusing on neurological and traumatic findings is important to evaluate for the subtle stroke, seizure, or traumatic brain injury.^1^,^5^,^6^,^8^–^10^

If the history and physical examination do not immediately elucidate the cause of AMS, the acronym AEIOU TIPS (Alcohol, Epilepsy/Electrolytes, Insulin/Inborn Errors of Metabolism, Overdose/Oxygen, Uremia, Trauma, Infection, Psychiatric/Poisoning, Stroke/Subarachnoid Hemorrhage (SAH)/Shock) can be used to consider a broader differential.^11^

#### Current Prehospital Treatment Recommendation

##### Level A Recommendation

- In a patient with AMS, obtain history of present illness, past medical history and cause for past episodes of AMS from patient or caregiver.
- A thorough physical examination is needed on all patients with AMS.

##### Level B Recommendation

- EMS should examine the scene for any evidence as to the cause of AMS (e.g., toxins) and communicate this finding to receiving personnel

##### Level C Recommendation

- To evaluate for the etiology of AMS, consider using the acronym AEIOU TIPS to provide a differential.

#### Clinical Question

What point-of-care tests should EMS perform on patients with AMS?

#### Summary of Current Evidence

Apart from a study in Finland,^8^ most research on causes of AMS focused on patients seen in the ED, rather than in the prehospital setting. However, from this information, we can deduce the possible causes of AMS in the prehospital setting, as many of these patients are brought to the ED by EMS, and can infer probable point-of-care tests that would be helpful. In this review, we define point-of-care tests as bedside testing, or diagnostic testing at the time of patient assessment.

In several studies, patients with an abnormal GCS were found to be more likely to have a history of conditions known to be associated with their current altered state, the most common of those being neurologic, toxicologic, diabetic-related, and infection.^5^,^6^,^8^,^9^ Hypoglycemia is one of the most common causes of AMS in adult patients in the prehospital setting; thus, rapid glucose testing is recommended for patients with AMS.^8^,^10^ Upon literature review, other point-of-care tests that have been evaluated for the use of evaluating AMS were 12-lead electrocardiogram (ECG), pulse oxygen (O_2_)-oximetry, pulse carbon monoxide (CO) oximetry, and end-tidal carbon dioxide detection (ETCO_2_).

Several studies demonstrated cardiac etiologies of AMS in the general population were infrequent, suggesting that a routine 12-lead ECG would not be helpful.^1^,^5^–^9^,^12^ However, if a dysrhythmia was noted on the cardiac monitor, obtaining a 12-lead ECG was useful to clarify the rhythm.^9^ In populations aged 65 years and older, there is a higher prevalence of cardiac causes of AMS, such as myocardial infarction (MI), complete heart block. 5,6 This suggests that for the elderly population with AMS there may be a benefit in obtaining a 12-lead ECG. Lastly, if an overdose is suspected with medications known to cause cardiac toxicity, such as antipsychotics, sodium channel blockers (tricyclic antidepressants ([TCAs]), diphenhydramine, beta-blockers (BB) and calcium channel blockers (CCB), consider obtaining a 12-lead ECG.^13^–^15^

Another cause of AMS is hypoxia, especially in the elderly population, which can be evaluated with pulse oximetry and may be considered the fifth vital sign.^16^,^17^ A similar point-of-care test is the pulse CO-oximeter. When looking at studies that compared Rad 57 (a type of pulse CO-oximeter) to the gold standard blood test, the evidence was conflicting, with wide ranges of precision and accuracy found.^18^–^20^ Since CO poisoning is not a common cause of AMS and since pulse CO-oximeter’s clinical accuracy remains unclear, we do not currently recommend evaluating for CO poisoning in the undifferentiated AMS patient.

Hypercapnia is a well-known cause of AMS. It is commonly observed with exacerbation of chronic obstructive pulmonary disease (COPD) and status asthmaticus, but may also be associated with pulmonary edema, neuromuscular respiratory failure, central hypoventilation, aspiration, and obesity hypoventilation syndrome.^21^,^22^ To evaluate hypercapnea in the field, ETCO_2_ is available. However, some researchers demonstrated a strong correlation between the gold standard PaCO_2_ and ETCO_2_^23^ while others have only demonstrated a correlation in the healthy state.^24^,^25^ It is our opinion that the causes of high and low ETCO_2_ measurements appear to be too numerous and complex to apply in the field for undifferentiated AMS at this time. However, extremes of measurement such as a high measurement >80mmHg would usually indicate high PaCO_2_.^22^ This would be a change in how ETCO_2_ is used in the prehospital setting since currently it is measured in those receiving positive pressure ventilation.^24^,^26^,^27^

Of note, breathalyzers, urine drug screens and lactate might be useful in some systems, but no prehospital studies on the use of these tests to evaluate patients for AMS were found during this literature review and they are not currently allowable for field use by paramedics in California.

#### Current Prehospital Treatment Recommendation

##### Level A Recommendation

- Place all patients with AMS on a cardiac monitor.
- Obtain pulse oximetry on all patients with AMS.
- Check blood glucose on every patient with AMS.
- Consider evaluating for a cardiac cause of AMS in the patient 65 years or older with a history of present illness or past medical history that suggests cardiac etiology.

##### Level B Recommendation

- Consider obtaining a 12-lead ECG on patients with AMS if they have a history of possible ingestion/overdose/intoxication, have an abnormal rhythm strip.

##### Level C Recommendation

- Not given

### General treatment for AMS

#### Clinical Question

What treatment is recommended in the prehospital setting when no cause of AMS has been identified?

#### Summary of Current Evidence

Most of the literature on AMS in the field and ED focuses on identifying the etiology. Once the cause is identified, the provider will implement the treatment pathways based on that assessment. Therefore, upon literature review, no evidence was found for a universal treatment that is appropriate for every patient with AMS.

The empiric treatment of AMS with a “coma cocktail” has largely been abandoned. This cocktail included one or more of the following medications: dextrose, naloxone, thiamine, and flumazenil. These medications are not without risk, so a more focused approach to treatment is required.^28^,^29^

#### Current Prehospital Treatment Recommendation

##### Level A Recommendation

- Not given

##### Level B Recommendation

- Not given

##### Level C Recommendation

- The empiric treatment of undifferentiated AMS with a “coma cocktail” should not be used.

### Supplemental Oxygen

#### Clinical Question

Should patients with AMS in the prehospital setting be treated with supplemental oxygen?

#### Summary of Current Evidence

Hypoxia can be detrimental to patients; even in healthy volunteers with <90% readings on pulse oximetry, the middle cerebral artery dilates.^30^ Hypoxia should be treated in a stepwise manner, with a goal of maintaining oxygen saturation ≥94%.^31^,^32^ Care should be taken to prevent hyperoxia because this can also be detrimental. In healthy volunteers, providing 100% oxygen for 10–15 minutes was associated with a 20–30% decrease in cerebral blood flow.^33^

Specific complaints and diagnoses that have historically led to the administration of empiric oxygen can result in worse outcomes when hyperoxia occurs. These include MI, dyspnea in COPD, and stroke. Hyperoxia may increase MI size, impair cardiac performance, and worsen heart failure.^34^–^36^ In COPD patients, hyperoxia can lead to hypercapnia, thus providing supplemental oxygen to keep saturations between 88% and 92% is recommended.^37^ Hyperoxia decreases cerebral blood flow from vasoconstriction and can increase ischemia in stroke and can decrease survival.^38^ In the setting of trauma, especially with traumatic brain injury, patients with significant hyperoxia (PaO_2_ >487) did worse.^39^,^40^

The surviving sepsis campaign guidelines also recommend that peripheral oxygen saturation be maintained between 88% and 95% in septic patients with adult respiratory distress syndrome, and advocate the avoidance of hyperoxia.^41^ In general, hyperoxia seems to impair oxygen delivery to patients during sepsis.^42^

#### Current Prehospital Treatment Recommendation

##### Level A Recommendation

- Provide supplemental oxygen to maintain O_2_ saturation ≥94%, unless COPD is present, then maintain a saturation of 88%–92%.
- Prevent hyperoxia in patients with MI, heart failure, stroke or COPD exacerbation.

##### Level B Recommendation

- Not given

##### Level C Recommendation

- Not given

### Use of a Standardized System or Score to Measure Level of Consciousness

#### Clinical Question

Is a standardized scoring system characterizing level of consciousness useful in the treatment of AMS in the prehospital setting?

#### Summary of Current Evidence

The GCS is the most widely used prehospital coma assessment tool. The GCS was originally developed to assess the head-injured patient, but has been adopted more broadly over the years to describe level of consciousness in patients with AMS of many etiologies, with subsequent studies suggesting that the GCS is valid in patients who are altered from toxicologic causes.^43^,^44^ Numerous studies have shown significant variability in inter-rater reliability for these scores, even among experienced physicians^45^–^47^ as well as more broadly across healthcare teams and inexperienced users.^48^–^50^ One study showed only moderate agreement between GCS determined in the prehospital setting and in the ED.^51^ The GCS is heavily weighted towards the motor score; therefore, low motor scores due to inability to cooperate may be misleading when predicting patient outcome particularly in patients with AMS.^52^

More recently, the Full Outline of UnResponsiveness (FOUR) score has been developed as an alternative to the GCS,^53^ with several studies showing this to be valid in both adults^54^–^56^ and children,^52^ while providing some additional prognostic information about brain stem injury. Most studies do not show a significant difference in inter-rater reliability between GCS and FOUR scoring systems.^54^,^56^,^57^

Another score that is used frequently in the prehospital setting is AVPU (awake, verbal stimuli, painful stimuli, and unresponsive/unconscious). This was introduced as a tool for rapid assessment of trauma patients as part of the Advanced Trauma Life Support course,^58^ with good correlation to GCS.^59^

#### Current Prehospital Treatment Recommendation

##### Level A Recommendation

- Not given

##### Level B Recommendation

- Choose a standardized scoring system, such as GCS or FOUR scale to assess level of consciousness in the prehospital setting for patients with AMS.
- The AVPU score can be used for rapid assessment of alertness, since it correlates well with GCS.

##### Level C Recommendation

- Not given

### Evaluate for Seizure

#### Clinical Question

Are patients with AMS in the prehospital setting having a seizure or are they in postictal phase?

#### Summary of Current Evidence

Numerous studies demonstrate that seizures are one of the most common causes of AMS. 1,6,8–10,60 When a patient exhibits obvious seizures, a seizure protocol will be implemented by paramedics instead of an AMS protocol. It is more challenging to identify prolonged postictal states, non-convulsive status epilepticus (NCSE) and partial seizures, which are all seen more frequently in elderly and pediatric populations.^5^,^10^

Most studies that examine seizures in the emergency setting do not indicate if the seizure was obvious, difficult to identify, or later identified to be NCSE. However, a study in 2014 by Zehtabchi assessed rates of NCSE confirmed with EEG and found undifferentiated altered patients had a 5% chance of being in NCSE.^61^ NCSE can present with discrete and subtle muscle twitching of face or limbs, increased tone, automatisms, clonic jerks, eye deviations/twitching, repetitive behaviors or prolonged postictal phase.^62^,^63^

#### Current Prehospital Treatment Recommendation

##### Level A Recommendation

- Not given

##### Level B Recommendation

- Consider treating for non-convulsive or subclinical seizures with history of previous episodes or prolonged postictal state, focal muscle twitching, automatisms, clonic jerks, eye deviations or repetitive behaviors.

##### Level C Recommendation

- Not given

### Evaluate for Trauma

#### Clinical Question

What factors make traumatic brain injury the likely cause for AMS in the prehospital setting?

#### Summary of Current Evidence

Most studies excluded obvious trauma while evaluating patients with AMS. Some patients with AMS were found to have occult traumatic brain injury (TBI). Otherwise occult trauma was not found to be a major cause of AMS.^5^,^7^,^8^ If intoxication is present, especially from alcohol, the evaluation is more challenging and less accurate. Due to alcohol use, these patients as well as elderly patients and those on anticoagulation or antiplatelet therapy are at higher risk for occult TBI, especially intracranial hemorrhage.^64^–^67^

#### Current Prehospital Treatment Recommendation

##### Level A Recommendation

- Not given

##### Level B Recommendation

- Consider TBI in patients with undifferentiated AMS, especially in the setting of intoxication, anticoagulation or antiplatelet therapy and in the elderly.

##### Level C Recommendation

- Not given

### Treatment of Hypoglycemia

#### Clinical Question

When and how should EMS providers treat hypoglycemia in patients with AMS?

#### Summary of Current Evidence

There is significant variation in how hypoglycemia is treated. About 12% of hypoglycemic patients present with AMS.^68^ Many EMS systems and EDs are switching from using dextrose 50% (D50) to dextrose 10% (D10). Seventy percent of agencies in the U.S. as of 2016 only allowed D50 for the treatment of hypoglycemia in adults, 8% only D10, and 22% either D10 or D50 with a trend toward increasing use of D10.^69^ This transition to D10 use is occurring for several reasons, including less extravasation risk, less acute hyperglycemia, less rebound hypoglycemia, and shortages of D50. D10 is less expensive and can be used in every age group. Many studies have demonstrated the feasibility, safety, and efficacy of using D10 instead of D50, with no increased time to resolution of symptoms and no significant differences in on-scene times.^70^,^71^ In comparing glucagon intramuscular (IM) to dextrose intravenous (IV), median time to full orientation for glucagon was 10–30 minutes, compared with 1–10 minutes for dextrose.^72^,^73^

The median blood glucose level threshold for treatment of hypoglycemia was 60mg/dL for patients of all ages.^69^ It is the committee’s opinion to treat hypoglycemia at 60 mg/dL in an adult. However, if clinically indicated hypoglycemia may be treated at higher levels in diabetic patients. The most frequently specified initial dose of glucose was 25gm of glucose for adults and 0.5 g/kg for pediatric patients.^69^

#### Current Prehospital Treatment Recommendation

#### Level A Recommendation

- Not given

##### Level B Recommendation

- In patients with AMS and hypoglycemia treat with oral glucose if indicated, or if venous access is available administer IV dextrose; IM glucagon is a second line agent.
- The preferred medication for treatment of AMS due to hypoglycemia is D10; if not available, D25 or D50 may be substituted.

##### Level C Recommendation

- Not given

### Evaluate for Toxicologic Causes of AMS

#### Clinical Question

How should patients in the prehospital setting be evaluated and treated for toxicologic causes of AMS?

#### Summary of Current Evidence

Toxicologic causes of AMS are common and result from a large number of toxins. The result is often a marked reduction in GCS.^9^ However, in patients >65 years old, toxicologic causes of AMS are less frequent.^5^ A history of depression, medication use, or illicit substance ingestion, especially alcohol, are important risk factors for a toxicologic cause of AMS. Almost 50% of alcohol-intoxicated patients who present to the ED arrive by ambulance and have higher blood alcohol levels and lower GCS scores than those arriving via private means.^64^

Drugs like methylenedioxymethamphetamine (MDMA), gamma-hydroxybutyrate (GHB), and synthetic cannabinoids are gaining popularity, especially by persons visiting clubs and festivals. Of those patients who seek medical help after GHB, most are altered, some with severely depressed GCS ≤9. Hallucinations, hypotension, bradycardia/tachycardia and hypo/hyperthermia are commonly found. Cooling measures, IV fluids, and symptomatic support including benzodiazepines are treatments that may be indicated for agitated delirium or seizures in this setting.^74^,^75^

If sodium channel blocker overdose is suspected (e.g., following diphenhydramine or TCA ingestions), sodium bicarbonate may be given. For calcium channel blocker (CCB) and beta blocker (BB) overdoses, calcium gluconate/chloride and glucagon are appropriate. These interventions have been demonstrated to be safe in the ED,^76^,^77^ but have not been studied in the prehospital setting.

#### Current Prehospital Treatment Recommendation

##### Level A Recommendation

- Not given

##### Level B Recommendation

- If an amphetamine or another sympathomimetic is ingested, treat symptomatically with cooling, IV fluids and benzodiazepines as needed.
- If a sodium channel blocker ingestion/overdose is suspected in an altered patient, consider sodium bicarbonate administration.
- If a CCB or BB ingestion/overdose is suspected in an altered patient, consider giving calcium and/or glucagon.

##### Level C Recommendation

- Not given

### Naloxone for Opioid Overdose

#### Clinical Question

When should naloxone be administered in the prehospital setting in patients with AMS?

#### Summary of Current Evidence

Treating patients with AMS empirically with naloxone is of no benefit unless there is evidence of opioid ingestion with respiratory depression. However, if there is concern for opioid overdose, naloxone has proven to be relatively safe. Naloxone has been found to be associated with a small but consistent rate of complications like seizures, arrhythmias, and severe agitation.^78^–^81^

Most of the criteria that studies examined when considering opioid overdose were respiratory rate ≤12, pinpoint pupils, and presence of drug paraphernalia, with AMS. These were found to be highly sensitive in predicting a response to naloxone. Miotic pupils outperformed respiratory rate as the best single criterion, with 91% sensitivity.^82^–^84^

To protect EMS personnel, several studies compared various routes of naloxone administration. IV, IM and intranasal (IN) administration of naloxone all result in reversal of opioid-overdose symptoms.^85^ IN naloxone is statistically as effective as IV and IM naloxone, causes improvement and withdrawal effects almost as rapidly as IV, but requires rescue doses more often.^86^–^89^ IN naloxone was shown to be faster, easier to administer and perceived as safer in those trained.^90^ This evidence suggests that IN is the preferred route, with IV and IM as alternative routes.

#### Current Prehospital Treatment Recommendation

##### Level A Recommendation

- Administer naloxone IN for AMS patients with evidence of hypoventilation, (i.e., respiratory rate ≤12), pinpoint pupils, presence of drug paraphernalia, and AMS.

##### Level B Recommendation

- Do not empirically administer naloxone without a clinical suspicion of opioid ingestion/overdose.
- Alternative routes for naloxone administration are IM or IV routes.

##### Level C Recommendation

- Not given

### Pediatric Altered Mental Status

#### Clinical Question

How are the causes of pediatric AMS different from those of an adult?

#### Summary of Current Evidence

The recommendations listed previously for adults apply for children as well, except for the recommendation to obtain a 12-lead ECG. Cardiac causes of AMS are exceedingly rare in children, so a 12-lead ECG is unlikely to be useful unless a dysrhythmia is suspected or evident on a rhythm strip. Studies did reveal that seizures, shock (e.g., sepsis), apparent life-threatening event (ALTE), now called brief resolved unexplained events (BRUE), hypoglycemia, and electrolyte abnormalities are common causes for pediatric AMS.^10^,^91^

Hypoglycemia can be seen in children for the same reasons as in adults, but pediatric patients are also at higher risk of hypoglycemia from toxic ingestions (e.g., ethanol), dehydration and sepsis.^10^ While the blood glucose level that requires treatment of hypoglycemia in children is variable, many EMS systems have used < 60 mg/dL universally for all patients.

TBI is another cause of AMS in children, especially non-accidental trauma.^10^,^91^,^92^ Although strokes are not usually considered a common pediatric cause of AMS, they do occur and their presentations are delayed because the diagnosis of stroke in children is often unrecognized.^93^

ED chart reviews identified common pediatric toxicologic emergencies causing AMS that require resuscitation including ingestion of ethanol, clonidine and acetaminophen. Other toxins more rarely causing AMS in children include CCB, BB and TCA.^76^,^77^,^91^,^94^

#### Current Prehospital Treatment Recommendation

##### Level A Recommendation

- Consider toxicologic causes as history and physical examination dictate and treat with naloxone if opioid ingestion is suspected in the setting of respiratory depression.

##### Level B Recommendation

- Place all pediatric patients with AMS on a cardiac monitor.
- Obtain pulse oximetry on all pediatric patients with AMS.

##### Level C Recommendation

- Check blood glucose on every pediatric patient with AMS and treat symptomatic hypoglycemia at values less than 60 mg/dL.

## RESULTS

We reviewed protocols from all 33 EMS agencies within California for consistency with the recommendations made by EMDAC for prehospital AMS management (Tables 1–3). Of the 33 LEMSAs, 30 (91%) have specific AMS protocols, often named “Altered Level of Consciousness.”

### Point-of-Care Tests

All LEMSAs recommend evaluation of blood glucose as part of their AMS protocols. Twenty-seven percent recommend obtaining a 12-lead ECG for adult patients with AMS, while other LEMSAs only recommend placing the patient on a cardiac monitor.

### General Treatment of AMS

No LEMSA suggests empiric treatment of AMS with dextrose, glucose, glucagon or naloxone without evidence of hypoglycemia or concern for opioid overdose.

### Supplemental Oxygen

The most common recommendation is providing supplemental oxygen (48% of LEMSAs) as needed. Only seven (21%) agencies provide parameters for oxygen supplementation. Three (9%) recommend general high-flow oxygen for all patients, and seven (21%) do not mention supplemental oxygen in the protocol itself.

### Use of a Standardized System or Score to Measure Level of Consciousness

Thirteen (39%) of the LEMSAs mention GCS in their protocols, often guiding the prehospital care provider to use the AMS protocol when the GCS <15.

### Evaluation for Seizure

Nineteen (58%) of the LEMSAs suggest evaluating for seizure as a cause of AMS. Many of these systems use the acronym AEIOU TIPS to allow for this consideration.

### Evaluation for Trauma

The majority of LEMSAs (79%) recommend evaluating the patient with AMS for signs of trauma.

### Evaluation for Hypoglycemia

The majority of LEMSAs (67%) suggest treating at <60mg/dL, the other levels recommended for treatment are <70, <75, and <80 mg/dL. More than half the agencies (64%) use D10 to treat symptomatic hypoglycemia while the remainder use D50. There is a trend away from D50 at this time. The most common suggested first dose of dextrose for adults is 25gm (73%), though there is a trend toward smaller initial doses.

### Evaluation for Toxicologic causes of AMS

Seventeen (52%) of the LEMSAs suggest evaluating for toxicologic causes of AMS, often by scene assessment and history from bystanders.

### Naloxone for Opioid Overdose

Forty-two percent of the LEMSAs provide specific parameters for naloxone administration, whereas 19 (58%) advise naloxone administration if opioid overdose is likely. Of agencies that recommend specific parameters, most provide a respiratory rate below which naloxone should be administered, the most common being ≤12 breaths per minute. In terms of the route of naloxone administration, 28 agencies (85%) allow IV, IM, or IN.

### Pediatric Altered Mental Status

Twenty-four (73%) of the LEMSAs have a different protocol for pediatric AMS than for adults. Of the 30% that do not provide a separate document, 21% provide pediatric recommendations in parallel to those for adults on the same document. One agency simply refers to the pediatric drug card.

## CONCLUSION

A wide range of disease processes can cause AMS. Because of the rapid treatment needed for many of these causes, prompt identification is important. Though few studies address specific assessment and treatment recommendations for AMS in the prehospital setting, we have ED studies that can be extrapolated for use prehospital, although not ideal. The evidence-based recommendations presented in this paper will inform EMS medical directors and guide creation of protocols for identifying and treating patients presenting with undifferentiated AMS in the prehospital setting.

## Supplementary Information



## Figures and Tables

**Figure:**
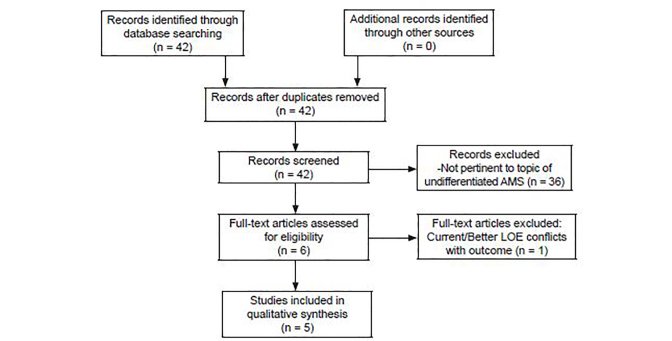
Preferred Reporting Items for Systematic reviews and Meta-Analyses (PRISMA) diagram based on initial PubMed search term “Prehospital and Altered Mental Status”. *AMS,* altered mental status. *LEMSA*, Local EMS Agency; *PED*, Pediatric; *D10*, Dextrose 10%; *D25*, Dextrose 25%; *D50*, Dextrose 50%; *NA*, not applicable; *IM*, intramuscular; *IN*, intranasal; *SC*, subcutaneous; *yo*, year old; *mo*, months.

**Table 1:** Treatment of hypoglycemia in adults and pediatrics.

LEMSA	Dextrose used	Level treated (mg/dL)	Dextrose dose (gm)	PED D10		PED D25		PED D50		Glucagon	
				
	For adult	For adult	For adult: 1st,2nd	D10 age	D10 dose (gm/kg)	D25 age	D25 dose (gm/kg)	D50 age	D50 dose (gm/kg)	Adult (mg)	Pediatrics
Alameda	D10	<60	10,15	All	0.2 <30 sec, 0.5	NA	NA	NA	NA	1 IM	0.1mg/kg IM
Central California	D50	<80	25,25	NA	NA	<2yo	0.5	>2yo	0.5	1 IN, IM	Per Broselow Tape
Coastal Valleys	D50/D10	<60–80	15,10	All	0.2 neonate, 0.5	NA	NA	NA	NA	1 IM	1mg IM
Contra Costa	D10	<60	10,15	All	0.5	NA	NA	NA	NA	1 IM	0.5, 1mg >24 kg IM
El Dorado	D10	<60	10,10	All	0.2<1mo, 0.5	NA	NA	NA	NA	1 IM,IN	0.1mg/kg IM,IN
Inland Counties EMS	D10	<60	25	All	0.5	NA	NA	NA	NA	1 IM,IN,SC	0.025mg/kg IM,IN
Imperial	D50	<60	25	NA	NA	0–2yo	0.5	>3yo	0.5	1 IM	0.05mg/kg IM
Kern	D10	<60	25,25	All	0.5	NA	NA	NA	NA	1 IM,IN	0.5mg, 1mg >8yo IM,IN
Los Angeles	D10	<60	12.5,12.5	All	0.5	NA	NA	NA	NA	1 IM	0.5mg, 1mg >1yo
Marin	D10	<60	12.5,12.5	All	0.2 neonate, 0.5	NA	NA	NA	NA	1 IM	0.3mg/kg IM
Merced	D50	<75	25,25	neonate	0.3	neonate-2yo	0.5	>2yo	0.5	1 IM	1mg IM
Monterey	D50	<70	25,25	neonate	0.2	>neonate	0.5	NA	NA	1 IM	0.5mg, 1mg >20kg IM
Mountain Valley	D50	<60	25,25	NA	NA	0–2yo	0.5	>2yo	0.5	1 IM	0.05mg/kg IM
Napa	D10	<60	25,5 q 5min	All	0.5	NA	NA	NA	NA	1 IM	1mg IM
North Coast	D50	<80	25	neonate	0.5–1	>neonate	1–2	NA	NA	1 IM	1mg IM
Northern California	D50	<75	25,25	neonate	0.5	>neonate	0.5	NA	NA	1 IM	1mg IM
Orange	D10	<60	25	neonate	0.5	neonate-1yo	0	>2yo	0.5	1 IM	0.5mg IM
Riverside	D50/10	<80	25	neonate	0.5	neonate-13kg	0.5	>14kg	0.5	1 IM	0.5mg, 1mg >14kg IM
Sacramento	D50/D10	<60	25,25	NA	NA	All	0.5	NA	NA	1 IM	0.5mg IM
San Benito	D10	<70	25,25	All	0.5	NA	NA	NA	NA	1 IM	0.5mg, 1mg >20kg IM
San Diego	D50	<60	25	All	1	All	0.5	NA	NA	1 IM	0.05mg/kg IM
San Francisco	D50	<60	25,25	<1mo	0.2	>1mo	0.5	NA	NA	1 IM	0.5mg, 1mg>20 kg
San Joaquin	D50/D10	<60	25,25	neonate	0.3	NA	NA	>neonate	0.25, 0.5 >2yo	NA	NA
San Luis Obispo	D50	<60	25	NA	NA	All	0.5	NA	NA	1 IM	0.1mg/kg IM
San Mateo	D50/10	<80	25,50/10,15	All	0.5	NA	NA	NA	NA	1 IM	0.5mg, 1mg >18kg
Santa Barbara	D10	<60	25,25	All	0.5	NA	NA	NA	NA	1 IM	0.1mg/kg IM
Santa Clara	D10	<80	10,20	All	0.3	NA	NA	NA	NA	1 IM	0.5, 1mg>6yo
Santa Cruz	D10	<70	25,25	All	0.5	NA	NA	NA	NA	1 IM	0.5mg, 1 mg>20kg
Sierra-Sacramento Valley	D50	<60	25	All	0.5	NA	NA	NA	NA	1 IM	0.5mg, 1mg>24kg
Solano	D50	<60	25,25	neonate	0.3	neonate-1yo	0.5	>2yo	0.5	1 IM	1mg IM
Tuolumne	D10	<75	25–50	All	0.5	NA	NA	NA	NA	1 IM	0.05mg/kg IM,IN
Ventura	D50/D10	<60	12.5,12.5/10,15	All	0.5	<2yo	0.5	>2yo	0.5	1 IM	0.1mg/kg IM
Yolo	D10	<60	25	All	0.5	NA	NA	NA	NA	1 IM	0.5mg IM,IN

*LEMSA*, Local EMS Agency; *PED*, Pediatric; *D10*, Dextrose 10%; *D25*, Dextrose 25%; *D50*, Dextrose 50%; *NA*, not applicable; *IM*, intramuscular; *IN*, intranasal; *SC*, subcutaneous; *yo*, year old; *mo*, months.

**Table 2:** Naloxone criteria and suggested dose.

LEMSA	Trigger		Dose	
	
	Adult	Pediatrics	Adult (mg)	Pediatrics
Alameda	RR<8	RR<12	1–2 IV,IM,IN	0.1mg/kg IV,IM
Central California	RR<8	NA	1 IV,IM, 2 IN	0.1mg/kg IV,IM,IN
Coastal Valleys	NA	NA	1–2 IV,IM,IN	0.1mg/kg IV,IM,IN
Contra Costa	RR<8	NA	1–2 IV,IM,IN	0.1mg/kg IV,IM
El Dorado	NA	NA	0.5–2 IV,IM,IN,ET	0.1mg/kg IV,IM,IN
Inland Counties EMS	NA	NA	0.5–10 IV,IM,IN	0.1mg/kg, 0.5–10mg >8yo IV,IM,IN
Imperial	RR <12	NA	0.5–2 IV,IM,IN	0.1mg/kg IV,IM
Kern	NA	NA	0.4–2 IV,IM,IN	0.1mg/kg,2mg >5yo IV,IM,IN
Los Angeles	NA	NA	0.8–4 IV,IM,IN	0.1mg/kg IV,IM,IN
Marin	NA	NA	0.4–2 IV,IM,IN	0.1mg/kg IV,IM,IN
Merced	NA	NA	1–2 IV,IM	2mg IV, IM, ET
Monterey	RR<10	RR<10	2 IV,IM,IN	0.1mg/kg IV,IM,IN
Mountain Valley	RR<10, SBP <90	NA	2 IV,IM,IN	0.1mg/kg IV, IM, IN
Napa	NA	NA	2 IV,IM,IN	0.4–2mg IV,IM,IN
North Coast	NA	NA	0.4–2 IV,IM,IN	0.01mg/kg IV, IM, IN
Northern California	NA	NA	0.4–6 IV,SQ,IM,IN	0.1mg/kg IV,IM,IN
Orange	RR<12	RR<12	0.4–2 IV, IM, IN	0.1mg/kg IV,IM,IN
Riverside	NA	NA	2 IV,IM,IN	0.1mg/kg IV,IM,IN
Sacramento	RR<16	NA	1–6 IV,IM,IN	0.1mg/kg IV,IM,IN
San Benito	NA	NA	0.5–2 IV,IM,IN	0.01mg/kg IV,IM,IN
San Diego	<12	NA	2 IV,IM,IN	0.1mg/kg IV,IM,IN
San Francisco	NA	NA	0.4–2 IV,IM,IN	0.1mg/kg IV,IM,IN
San Joaquin	NA	NA	1–2 IV,IM,IN	0.1mg/kg IV,IM,IN
San Luis Obispo	RR<94%, ETCO2>45	NA	0.4–2 IV,IM,SL	0.4–2mg IV,IM,IN
San Mateo	NA	NA	1–2 IV,IM	0.1mg/kg IV,IM
Santa Barbara	<12	<12	0.4–2 IV,IM,IN	0.1mg/kg IV,IM,IN
Santa Clara	NA	NA	1–2 IV,IM	0.1mg/kg IV,IM
Santa Cruz	NA	NA	0.5–2 IV,IM,IN	0.01mg/kg IV,IM,IN
Sierra-Sacramento Valley	<12	Inadequte RR	1–2 IV,IM,IN	0.1mg/kg IV,IM,IN
Solano	<8	NA	0.5–2 IV,IM,IN	0.5–2mg IV,IM
Tuolumne	NA	NA	0.4–2 IV,IM,IN	0.1mg/kg IV,IM
Ventura	<12	<12	0.4–2 IV,IM	0.1mg/kg IV,IM
Yolo	NA	NA	2 IV,IM,IN	0.1mg/kg IV,IM

*LEMSA*, Local EMS Agency; *RR,* respiratory rate; *SBP*, systolic blood pressure; *IV*, intravenous, *NA*, not applicable; *IM*, intramuscular; *IN*, intranasal; *ET,* endotracheal tube; *yo*, year old; *mo*, months.

**Table 3:** Evaluating patients for various etiologies of altered mental status (AMS).

LEMSA	Separate PEDS protocol	EKG	Supplemental O_2_	Use of GCS	Assess for trauma	Assess for stroke	Assess for seizure	Assess for TOX except narcotics	How to consider differential
Alameda	Y	Y	<94%	N	Y	Y	Y	Y	AEIOU TIPS
Central California	N-same doc	N	High flow	N	Y	Y	Y	Y	AEIOU TIPS
Coastal Valleys	Y	Y	NA	Y	Y	Y	Y	Y	AEIOU TIPS
Contra Costa	N-same doc	Y	<94%	Y	Y	Y	Y	Y	AEIOU TIPS
El Dorado	Y	N	PRN	N	Y	N	Y	Y	List
Inland Counties EMS	Y	N	PRN	N	Y	N	Y	Y	List
Imperial	No-PEDS drug guide	N	<94%	N	Y	Y	Y	N	List
Kern	N-same doc	N	PRN	N	Y	N	Y	Y	List
Los Angeles	N-same doc	Y	PRN	N	Y	N	N	N	List
Marin	Y	N	NA	Y	Y	Y	Y	Y	AEIOU TIPS
Merced	Y	N	High flow	Y	Y	N	N	N	List
Monterey	Y	N	NA	N	N	N	N	N	No AMS protocol
Mountain Valley	Y	N	PRN	Y	N	N	N	N	NA
Napa	Y	Y	PRN	Y	Y	Y	Y	Y	No AMS protocol, AEIOU TIPS
North Coast	N-same doc	N	High flow	Y	N	Y	N	Y	List
Northern California	Y	N	<92%	Y	Y	Y	Y	N	List
Orange	Y	N	<95%	N	N	Y	N	N	List
Riverside	N	N	NA	N	Y	Y	Y	Y	AEIOU TIPS
Sacramento	Y	N	<94%	Y	Y	Y	Y	Y	AEIOU TIPS
San Benito	Y	N	NA	N	Y	N	N	N	List
San Diego	Y	Y	<94%	N	Y	Y	Y	N	List
San Francisco	Y	N	PRN	N	Y	N	N	N	List
San Joaquin	Y	N	NA	Y	Y	Y	N	Y	List
San Luis Obispo	Y	N	PRN	N	Y	Y	Y	Y	AEIOU TIPS
San Mateo	Y	N	PRN	N	Y	Y	Y	N	List
Santa Barbara	N-same doc	N	PRN	N	N	Y	N	N	List
Santa Clara	Y	Y	PRN	N	Y	Y	Y	Y	List
Santa Cruz	Y	N	NA	N	Y	N	N	N	List
Sierra-Sacramento Valley	Y	N	PRN	Y	N	N	N	N	List
Solano	N	N	PRN	N	Y	Y	N	N	List
Tuolumne	Y	Y	PRN	Y	N	N	N	N	List
Ventura	N-same doc	N	PRN	N	Y	Y	Y	Y	AEIOU TIPS
Yolo	Y	Y	PRN	Y	Y	Y	Y	Y	AEIOU TIPS

*LEMSA*, Local EMS Agency; *PEDS*, Pediatrics; *EKG*, electrocardiogram; *GCS*, Glasgow Coma Scale; *TOX*, toxicology; *Y*, yes; *N*, no; *AEIOU TIPS*, Alcohol, Epilepsy/Electrolytes, Insulin, Overdose/Oxygen, Uremia, Trauma, Infection, Psychiatric, Stroke/Subarachnoid Hemorrhage (SAH)/Shock; *doc*, document; *NA*, not applicable; *PRN*, as needed.
