# Supplementary material for: Altered Mental Status: Current Evidence-based Recommendations for Prehospital Care
Source: West J Emerg Med. 2018 Mar 8;19(3):527–41. doi: 10.5811/westjem.2018.1.36559 (PMC5942021; doi:10.5811/westjem.2018.1.36559)
Supplement: Supplementary file 1 [file wjem-19-527-s001.docx]

| **Clinical Question** | **Search Terms** |
| --- | --- |
| What patient and scene assessment should be performed by EMS for patients with AMS? | Prehospital/EMS, AMS/ALOC, patient assessment, scene assessment, scene safety |
| What point-of-care tests should EMS perform on patients with AMS? | Prehospital/EMS, AMS/ALOC, blood glucose/sugar, ECG/EKG, ETCO_2_, hypercapnea, point-of-care tests, pulse oximetry |
| What treatment is recommended in the prehospital setting when no cause of AMS has been identified? | Prehospital/EMS, AMS/ALOC, empiric treatment, universal treatment, treatment of |
| Should patients with AMS in the prehospital setting be treated with supplemental oxygen? | Prehospital/EMS, AMS/ALOC, hyperoxia, hypoxia, supplemental oxygen |
| Is a standardized scoring system characterizing level of consciousness useful in the treatment of AMS in the prehospital setting? | Prehospital/EMS, AMS/ALOC, AVPU, FOUR scale, GCS, level of consciousness, scoring system |
| Are patients with AMS in the prehospital setting having a seizure or postictal? | Prehospital/EMS, AMS/ALOC, non-convulsive seizures, prolonged postictal phase/state, status epileptics, subclinical seizures |
| What factors make traumatic brain injury the likely cause for AMS in the prehospital setting? | Prehospital/EMS, AMS/ALOC, alcoholism and trauma, antiplatelet/ anticoagulation and trauma, intoxication and trauma, occult trauma |
| When and how should EMS providers treat hypoglycemia in patients with AMS? | Prehospital/EMS, AMS/ALOC, blood glucose/sugar D10, D50, glucagon, hypoglycemia. |
| How should patients in the prehospital setting be evaluated and treated for toxicologic causes of AMS? | Prehospital/EMS, AMS/ALOC, ingestion, overdose, toxicologic causes |
| When should naloxone be administered in the prehospital setting in patients with AMS? | Prehospital/EMS, AMS/ALOC, naloxone, naloxone route, Narcan, opioid overdose |
| How are the causes of pediatric AMS different from those of an adult? | Prehospital/EMS, AMS/ALOC, child/children, pediatric |

**Appendix Table:** Search Terms Used for Clinical Questions.

Articles chosen based on relevance to the question being asked.

*EMS*, Emergency Medical Services; *AMS,* Altered Mental Status, *ALOC*, Altered Level of Consciousness; *EKG/ECG*, Electrocardiogram; *ETCO_2_*, End-tidal Carbon Dioxide; *AVPU*, Alert Voice Pain Unresponsive; *FOUR*, Full Outline of Unresponsiveness, *GCS*, Glasgow Coma Scale; *D10*, Dextrose 10%; *D50*, Dextrose 50%
